# Effects of Physiochemical Factors on Prokaryotic Biodiversity in Malaysian Circumneutral Hot Springs

**DOI:** 10.3389/fmicb.2017.01252

**Published:** 2017-07-06

**Authors:** Chia S. Chan, Kok-Gan Chan, Robson Ee, Kar-Wai Hong, María S. Urbieta, Edgardo R. Donati, Mohd S. Shamsir, Kian M. Goh

**Affiliations:** ^1^Faculty of Biosciences and Medical Engineering, Universiti Teknologi MalaysiaSkudai, Malaysia; ^2^Division of Genetics and Molecular Biology, Faculty of Science, Institute of Biological Sciences, University of MalayaKuala Lumpur, Malaysia; ^3^CINDEFI (CCT, La Plata-CONICET, UNLP), Facultad de Ciencias Exactas, Universidad Nacional de La PlataLa Plata, Argentina

**Keywords:** 16S rRNA amplicon sequencing, hot spring metagenome, saline pool, microbial community, thermophile diversity, microbial symbiosis, microbiome

## Abstract

Malaysia has a great number of hot springs, especially along the flank of the Banjaran Titiwangsa mountain range. Biological studies of the Malaysian hot springs are rare because of the lack of comprehensive information on their microbial communities. In this study, we report a cultivation-independent census to describe microbial communities in six hot springs. The Ulu Slim (US), Sungai Klah (SK), Dusun Tua (DT), Sungai Serai (SS), Semenyih (SE), and Ayer Hangat (AH) hot springs exhibit circumneutral pH with temperatures ranging from 43°C to 90°C. Genomic DNA was extracted from environmental samples and the V3–V4 hypervariable regions of 16S rRNA genes were amplified, sequenced, and analyzed. High-throughput sequencing analysis showed that microbial richness was high in all samples as indicated by the detection of 6,334–26,244 operational taxonomy units. In total, 59, 61, 72, 73, 65, and 52 bacterial phyla were identified in the US, SK, DT, SS, SE, and AH hot springs, respectively. Generally, Firmicutes and Proteobacteria dominated the bacterial communities in all hot springs. Archaeal communities mainly consisted of Crenarchaeota, Euryarchaeota, and Parvarchaeota. In beta diversity analysis, the hot spring microbial memberships were clustered primarily on the basis of temperature and salinity. Canonical correlation analysis to assess the relationship between the microbial communities and physicochemical variables revealed that diversity patterns were best explained by a combination of physicochemical variables, rather than by individual abiotic variables such as temperature and salinity.

## Introduction

The study of extremophiles provides insights into the origin and evolution of life. Biologists believe that extremophiles inhabiting extreme environments such as hot springs are the closest living descendents of the earliest life forms on earth (Woese et al., 1990; Olsen et al., 1994). Additionally, these extreme environments comprise relatively simple microbial ecosystems as compared with more complex environments such as soils (Xu et al., 2014), wastewater (Shanks et al., 2013), marine sediments (Zheng et al., 2014), and the human gastrointestinal tract (Trosvik and de Muinck, 2015). Therefore, studying the life in hot springs enables us to interrogate the interactions between organisms and the environment. Microbial communities in hot springs, especially those in Yellowstone National Park (USA) (Blank et al., 2002; Kan et al., 2011; Inskeep et al., 2013), Japan (Kubo et al., 2011; Nishiyama et al., 2013; Masaki et al., 2016), Iceland (Tobler and Benning, 2011; Menzel et al., 2015), China (Hou et al., 2013; Song et al., 2013), and India (Badhai et al., 2015; Sangwan et al., 2015), are extensively studied. Among these, the Octopus and Mushroom Springs at Yellowstone National Park have the longest history of research of nearly 50 years (Thiel et al., 2016). Recently, a research team in New Zealand initiated the 1,000 Springs Research Project to examine geothermal microbial biota in 1,000 hot-spring features in the Taupo Volcanic Zone^1^. Microbial populations in heated springs are influenced by physicochemical factors, such as temperature, pH, dissolved oxygen, and water chemistry (Mathur et al., 2007; Sharp et al., 2014; Chan et al., 2015). In hot springs with temperatures higher than 75°C, Aquificae, Deinococcus-Thermus, Thermodesulfobacteria, Thermotogae, and some thermophilic members of Proteobacteria and Firmicutes are the commonly found bacterial phyla (Blank et al., 2002; Hou et al., 2013; Song et al., 2013), in addition to archaeal phyla such as Crenarchaeota, Euryarchaeota, and Thaumarchaeota (Hou et al., 2013; Nishiyama et al., 2013; Chan et al., 2015). In hot springs with lower temperatures, thermophilic photosynthetic bacteria such as Cyanobacteria and Chloroflexi, together with phyla such as Proteobacteria may be the main population (Kubo et al., 2011; Nishiyama et al., 2013; Badhai et al., 2015). As the maximum temperature for photosynthesis is 75°C (Ferris and Ward, 1997), Cyanobacteria and Chloroflexi are therefore not predominantly present in hot springs with temperatures higher that this threshold.

The Banjaran Titiwangsa mountain range, with elevations of 900–2,100 m and a length of about 480 km, is the most prominent mountain cluster on Peninsular Malaysia. Most of the hot springs on the peninsula are located along the western flank of Banjaran Titiwangsa and are concentrated along major fault zones (Sum et al., 2010). The Malaysian hot springs differ from the geothermal systems in Yellowstone National Park, Japan, Kamchatka, and New Zealand in several aspects. Malaysia has a tropical climate and is located in a non-volcanic area. The water arising from these hot springs is heated geothermally; groundwater that percolates deeply into the Earth's crust comes in contact with the rocks that get heated as a result of the geothermal gradient originating from the Earth's interior. Pressure is generated and forces the water to discharge through pores and fissures within the Earth's crust toward the surface to form hot springs (Baioumy et al., 2015). In countries with volcanic activities in tectonically active zones, rain or melted snow comes in contact with near subsurface magma that may heat the water sufficiently to form superheated water bodies. Acidic hot springs (pH <6.0) are often found in volcanic areas; however, hot springs in Malaysia are circumneutral or slightly alkaline. Hot-spring water usually has high concentrations of various elements owing to mineralization of dissolved solid elements from the adjacent areas. The composition of hot water is mainly determined by chemical interactions with reservoir rocks and rock-forming minerals along the ascent path, which may cause the spring water to be acidic or alkaline.

Earlier reports summarized the different physicochemical conditions in 46 known Malaysian hot springs (Samsudin et al., 1997; Sum et al., 2010). These hot springs have temperatures ranging from 36°C to 102°C, but predominantly lower than 80°C. Malaysian hot springs exhibit differences in physical appearance and chemical contents, which are expected to influence the microbial communities; nevertheless, limited studies have been conducted to understand the microbial diversity in Malaysian (Goh et al., 2011; Chan et al., 2015) and South-East Asian (Baker et al., 2001; Kanokratana et al., 2004; Huang et al., 2013) hot springs. In this report, a coordinated geochemical and molecular survey was conducted for six hot springs. Ulu Slim, Sungai Klah, and Dusun Tua were selected as these sites are the hottest geothermal springs in Malaysia. Ayer Hangat was chosen because it is the only saline hot spring in Malaysia. Semenyih and Sungai Serai hot springs were selected as they exhibit similar temperature (~45°C) and pool size and are located close to each other. This work utilized 16S rRNA gene markers to understand microbial diversity and community structure. We expected our findings to elucidate possible relationships with hot-spring physicochemical variables.

## Materials and methods

### Study sites and water physicochemical characteristics

**Figure 1:**
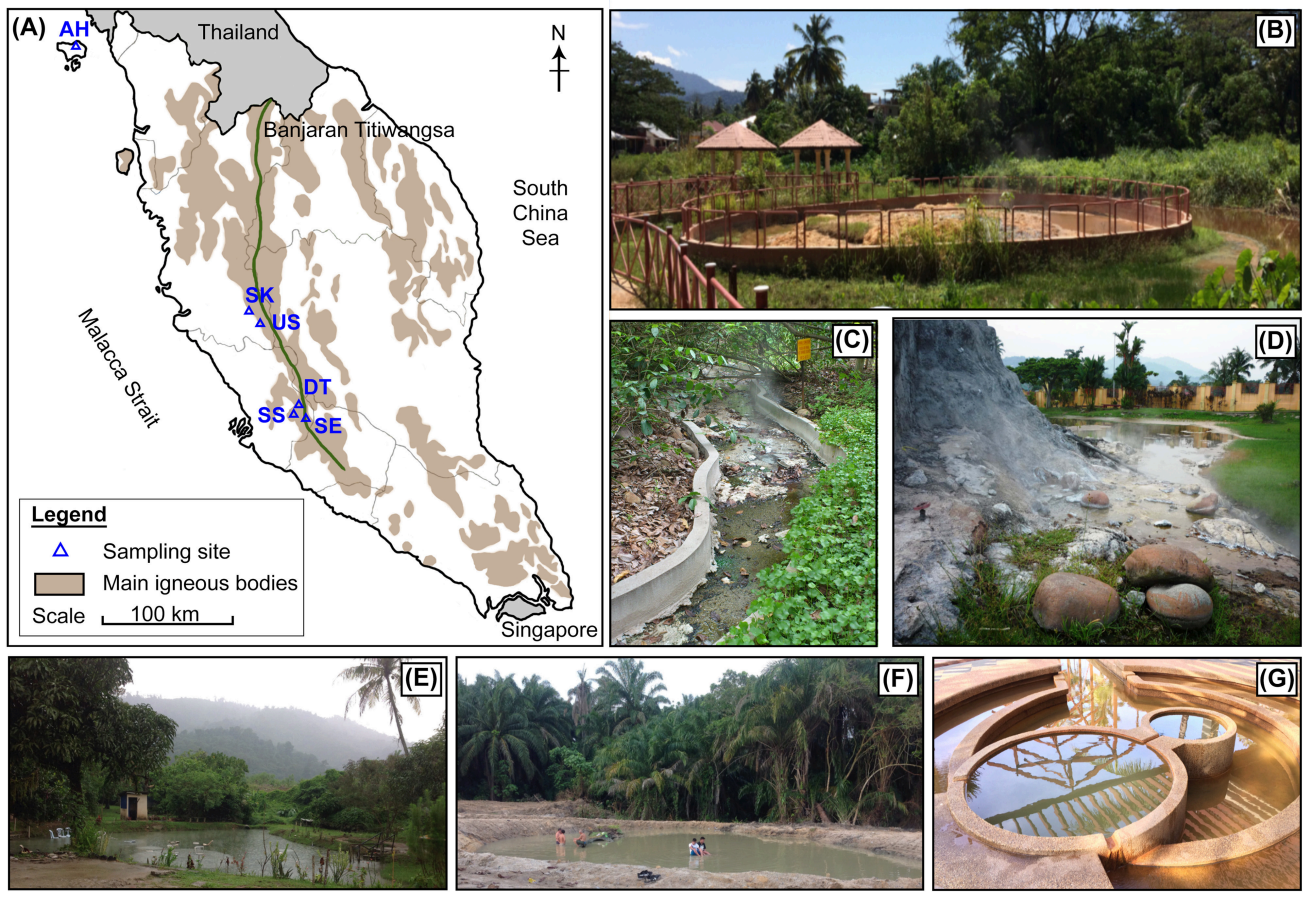
**(A)** Map of the locations of six selected hot springs in Malaysia: Ayer Hangat (AH), Ulu Slim (US), Sungai Klah (SK), Dusun Tua (DT), Sungai Serai (SS), and Semenyih (SE). Photographs of Malaysian hot springs sampling sites; **(B)** US, **(C)** SK, **(D)** DT, **(E)** SS, **(F)** SE, and **(G)** AH.

Hot springs at Ulu Slim (US) (3°53'55.79”N, 101°29'52.44”E), Sungai Serai (SS) (3°5'27.71”N, 101°47'39.06”E), Dusun Tua (DT) (3°8'21.23”N, 101°50'10.33'E), and Semenyih (SE) (3°2'32.81”N, 101°52'19.87”E) are located along the Banjaran Titiwangsa main mountain range (Figure 1). Additionally, the Ayer Hangat (AH) (6°25'22.31”N, 99°48'48.97”E) hot spring, located outside the main mountain range, was included in this study because of its high saline water content (Table 1). At each hot spring, water temperature was measured using a portable thermometer. Water and sediment samples were collected in sterile bottles that were closed immediately after sampling. Samples were collected in at least three sites at each hot spring. The samples were kept at ambient temperature and transferred immediately to the laboratory, where they were stored at 4°C prior to DNA extraction. Water samples of each site were sent to the Allied Chemists Laboratory Sdn. Bhd. (Malaysia) for physiochemical analyses in accordance with the American Public Health Association, Standard Methods for the Examination of Water and Wastewater (APHA) and United States Environmental Protection Agency (USEPA) guidelines (refer to Table 2 for the list of analyses).

**Table 1:** Descriptions of the hot springs studied.

**Name**	**Sample type^a^**	**Descriptions**
Ulu Slim (US)	Water and sediment (spring heads and pond)	• The hottest spring in Malaysia. • A roughly cylindrical shallow pool (~4 m diameter, ~10 cm depth) with two moderate spring heads (110°C). • Clear water and fine clays at the bottom. • Biofilms (<5 mm thickness) with colors of yellow, orange, light brown, and light green are formed on the spring.
Sungai Klah^b^ (SK)	Water and sediment (middle of stream)	• Shallow water flow stream (~150 m length, ~1.5 m width, ~5 cm depth) • Water temperatures between 50–110°C. • Multiple spring heads with clear running water. • Clays, rocks, and sands at the bottom.
Dusun Tua (DT)	Water and sediment (spring head and pond)	• A shallow pool with small spring source (75°C) outflow continually from a 1.5-m man-made cement-fountain landscape. • Multiple thick biomats (1–5 cm) of various colors (dark brown, red, white, dark green, and orange) are formed on the fountain, while a green filamentous mat is present on the bank of the collecting pool. • Clear water with silicate sands and clays at the bottom.
Sungai Serai (SS)	Water and sediment (center and edge of pool)	• Roughly round-shaped pool with diameter ~10 m, and depth ~0.5 m. • Many bubbling sources, but non-visible spring head. • Murky water with green biomats on the bank of the pool. • Loam soil at the bottom.
Semenyih (SE)	Water and sediment (center and edge of pool)	• Roughly round-shaped pool with diameter ~10 m, and depth ~0.7 m. • Many bubbling sources and no visible outflow. • Non-visible spring head. • Murky water with green biomats on the bank of the spring. • Fine silicate sands at the bottom.
Ayer Hangat (AH)	Water	• The only saline hot spring in Malaysia. • Water is trapped in a deep, cylindrical man-made pond with ~1-m diameter. • Formation of small degassing bubbles with slow outflow. • Gray-yellowish water mixed with fine salt. • Thin light-brown and green biomats (<1 cm) are formed on the surrounding wall.

a *Sample type used for genomic DNA extraction*.

b *Data collected from Diversity of thermophiles in a Malaysian hot spring determined using 16S rRNA and shotgun metagenome sequencing (Chan et al., 2015)*.

**Table 2:** Physicochemical properties of water samples from the six Malaysian hot springs studied.

**Test variable**	**Methods**	**Unit**	**US**	**SK^a^**	**DT**	**SS**	**SE**	**AH**
Temperature range	Thermometer	°C	80–110	60–110	55–75	40–45	40–50	40–50
Sampling temperature	Thermometer	°C	90	75	70	43	43	45
pH^b^	APHA 4500 H^+^ B		7.2	8.2	7.0	6.9	6.9	7.1
Alkalinity	APHA 2320 B	mg L^−1^	94	76	106	122	136	294
Acidity	APHA 2310 B	mg L^−1^	<1	<1	<1	<1	20	<1
Color	APHA 2120 B	TCU	<5	75	<5	<5	<5	5
Turbidity	APHA 2130 B	NTU	1.0	130	<0.05	1.0	0.5	1.1
C:N ratio (TOC/TN)^c^			1.5	1.6	0.1	0.3	0.1	5.0
Aluminum (Al)	APHA 3030 F/USEPA 6010 B	mg L^−1^	0.07	0.96	0.04	0.04	0.04	0.04
Arsenic (As)	APHA 3030 F/USEPA 6010 B	mg L^−1^	0.03	0.07	ND (<0.01)	ND (<0.01)	ND (<0.01)	ND (<0.01)
Chloride (Cl^−^)	APHA 4500-CI^−^ B	mg L^−1^	<1	2	1	3	4	13,832
Fluoride (F^−^)	APHA 4500-F^−^ D	mg L^−1^	0.4	1.1	6.9	11	9.4	21
Hardness (CaCO_3_)	APHA 2340C	mg L^−1^	13	<1	5	27	27	5,020
Iron (Fe)	APHA 3030 F/USEPA 6010 B	mg L^−1^	ND (<0.02)	0.65	ND (<0.02)	0.03	ND (<0.02)	ND (<0.02)
Magnesium (Mg^+^)	APHA 3030 F/USEPA 6010 B	mg L^−1^	0.8	0.5	<0.1	0.2	0.2	394
Phosphate (${\text{PO}}_{4}^{3-}$)	APHA 3030 G/USEPA 6010 B	mg L^−1^	0.1	0.2	0.1	0.3	0.7	0.4
Sodium (Na^+^)	APHA 3030 F/USEPA 6010 B	mg L^−1^	43	27	51	45	48	7,905
Sulfate (${\text{SO}}_{4}^{2-}$)	APHA 4500-SO_4_ E	mg L^−1^	3	8	6	1	1	947
Sulfur (S)	APHA 3030 F/USEPA 6010 B	mg L^−1^	5.2	3.9	2.7	0.5	1.1	477
TN	APHA 3030 F/USEPA 6010 B	mg L^−1^	<0.2	5.6	2.7	3.0	6.1	<0.2
TOC	APHA 5310 B	mg L^−1^	0.3	9.04	0.4	0.8	0.9	1.0
BOD 5 days at 20°C	APHA 5210 B	mg L^−1^	<5	5	<5	<5	<5	30
BOD 5 days at 60°C	APHA 5210 B	mg L^−1^	<5	10	<5	<5	<5	20
BOD 5 days at 80°C	APHA 5210 B	mg L^−1^	<5	5	<5	<5	<5	<5
COD	APHA 5220 B	mg L^−1^	7.4	35	<5	<5	<5	90

a *Data collected from Diversity of thermophiles in a Malaysian hot spring determined using 16S rRNA and shotgun metagenome sequencing (Chan et al., 2015)*.

b *The pH values were measured at room temperature*.

c *The C:N ratios were calculated on mass basis*.

*Abbreviations: APHA, in accordance with American Public Health Association, Standard Methods for the Examination of Water and Wastewater; USEPA, United States Environmental Protection Agency; TN, total nitrogen; TOC, total organic carbon; BOD, Biochemical oxygen demand; COD, Chemical oxygen demand; ND, not detected*.

### DNA extraction and 16S rRNA gene sequencing

DNA extraction was conducted in the laboratory. Equal volumes of water and sediment samples for each hot spring were collected and pooled to represent the overall microbiome of each site. DNA extraction and amplicon generation from environmental samples were conducted as previously described (Chan et al., 2015), with modification of the amount of starting material used for DNA extraction. In brief, genomic DNA extracted from the samples was amplified and dual-index barcoded for multiplex sequencing using primer pair (S-D-Bact-0341-b-S17 and S-D-Bact-0785-a-A-21) targeted to the V3–V4 regions of the 16S rRNA gene (Klindworth et al., 2013). The forward (5′-TCGTCGGCAGCGTCAGATGTGTATAAGAGACAG-CCTACGGGNGGCWGCAG-3′) and reverse (5′-GTCTCGTGGGCTCGGAGATGTGTATAAGAGACAG-GACTACHVGGGTATCTAATCC-3′) primers contained Illumina overhang adapter sequences (underlined regions). Amplicons were paired-end (2 × 300 bp, MiSeq v3 reagent) sequenced on an Illumina MiSeq sequencer (San Diego, CA, USA) at the High Impact Research Institute at the University of Malaya, Malaysia. The sequence data have been submitted to NCBI SRA under Bioproject accession number PRJNA378468. To compare microbial profiles obtained in this study with those reported earlier for the Malaysian SK hot spring (Chan et al., 2015), the SRA for the latter (Bioproject no. PRJEB7059) was retrieved and processed similarly to the reads generated in this work.

### Data analysis

The raw sequence data were evaluated and filtered to ensure that >80% of the base calls in a sequence had a Phred quality score of 20 using the FASTQ Quality Filter (*q* = 20, *p* = 80) of the FASTX-Toolkit^2^. Sequences that passed the quality filtering, were free from ambiguous characters, and of ≥200 bp were merged using PEAR (Zhang et al., 2014). Chimeric sequences were identified and discarded using the UCHIME algorithm (Edgar et al., 2011) implemented in the USEARCH package (Edgar, 2010). The sequences were then analyzed using the Quantitative Insights Into Microbial Ecology (QIIME) pipeline version 1.9.1 (Caporaso et al., 2010b) with default parameters unless otherwise noted. Briefly, sequences were clustered into operational taxonomic units (OTUs) at 97% similarity with USEARCH-based (Edgar, 2010) open-reference OTUs picking protocols using the Greengenes 13_8 reference sequences (McDonald et al., 2012). Taxonomy was assigned to sequences using UCLUST (Edgar, 2010), retrained on Greengenes 13_8 with a minimum of one OTU size, in QIIME. Representative sequences, which were selected as the centroid sequence of each OTU, were aligned using PyNAST aligner (Caporaso et al., 2010a) with a minimum sequence length of 26 bases, and those that failed to align were removed.

### Statistical analysis

Multivariate principal component analysis (PCA) of 23 physicochemical variables including sampling-site water temperature, pH, alkalinity, acidity, color, turbidity, aluminum, arsenic, chloride, fluoride, CaCO_3_, iron, magnesium, phosphate, sodium, sulfate, sulfur, total nitrogen (TN), total organic carbon (TOC), biochemical oxygen demand under 5-day incubation (BOD; at 20, 60, and 80°C), and chemical oxygen demand (COD) was carried out to determine which physicochemical variables were related to the observed hot spring community patterns. All measured physicochemical variables were checked for normality and log-transformed before PCA. A PCA plot was generated using PAST software version 3.13 (Hammer et al., 2001).

To estimate species richness from the data sets, the original OTU table with singletons was used to conduct rarefaction analysis and estimate alpha diversity. Rarefaction curves based on observed OTUs were generated using QIIME. The alpha diversity estimates, including Good's coverage, Shannon–Wiener's diversity index, and Simpson's index of diversity, were calculated. Sampling completeness was evaluated using the Good's average estimator, which calculates the probability that a randomly selected amplicon sequence from a sample has already been sequenced (Good, 1953). The Shannon–Wiener diversity index (Spellerberg and Fedor, 2003) was used to explain the entropy, taking into account the species richness and evenness of the community, which varied from 0 for communities with a single taxon, to high values for highly diverse communities. Simpson's index of diversity (1-D) (Simpson, 1949) was used to describe the diversity in a community, ranging from 0 to 1, with 1 indicating maximum diversity in a sample.

Singletons were discarded and the OTU table was rarefied to a depth of 313,337 sequences per sample (75% sequences to the lowest number of sequences found among the sample dataset) to minimize the effect of sampling effort. Further, diversity analyses including beta diversity analysis were conducted using QIIME, with the script core_diversity_analyses.py. Jackknifed unweighted pair group method with arithmetic mean (UPGMA) clustering was performed to compare microbial community similarity among the hot spring samples based on UniFrac phylogenetic distances (weighted and unweighted) (Lozupone et al., 2007) and non-phylogenetic Bray–Curtis dissimilarity distances. A relative small UniFrac distance implies that two communities are compositionally similar, harboring lineages that share a common evolutionary history. Unweighted UniFrac only accounts for the community membership, while weighted UniFrac accounts for community structure (membership and relative abundance) (Lozupone et al., 2007). Biplots of principal coordinate analysis (PCoA) were generated in QIIME and visualized in 3D using Emperor (Vázquez-Baeza et al., 2013).

Canonical correspondence analysis (CCA) was performed using PAST software, to explore relationships of microbial community patterns at the phylum level with physicochemical variables. By considering that predominant species have greater influence within the communities, only 75 major OTUs with relative abundance of >0.001% across all sample data sets were used as a community matrix for CCA. The significance of the CCA models and the explanatory factors were tested using 999 permutations. The ordination on the x- and y-axis and the length of the corresponding arrows indicate the relative importance of physicochemical variables explaining the taxon distribution across communities. Unless specified otherwise, all statistical analyses were done in QIIME and/or PAST (Hammer et al., 2001).

## Results

### Site descriptions and physicochemical characteristics of hot springs

In this study, six hot springs with different physical and chemical conditions were studied (Table 1). Basically, the selected sites could be categorized as: (1) pond with high NaCl content (AH hot spring), (2) fast-flowing streamer (SK hot spring), and (3) non-saline pool with standing water (US, DT, SS, and SE hot springs). Data on microbial diversity in SK were previously reported by our group (Chan et al., 2015) and were compared with data for the other five hot springs studied in the current work (Figure 1, Table 1). The six hot springs exhibit different temperatures (40–110°C) and pH (6.9–8.2). The highest temperature was recorded at US (80–110°C) followed by SK (60–110°C) and DT (55–75°C). Hot springs SS, SE, and AH have lower temperatures (40–50°C). The water chemistry including alkalinity, acidity, color, turbidity, and major ions varied among the hot springs (Table 2). In comparison to other sites, SK water has the highest turbidity, TOC, aluminum, arsenic, and iron, while AH had distinctly high concentrations of chloride, fluoride, CaCO_3_ (hardness), magnesium, sodium, sulfate, and sulfur. The chemical contents in water samples from high-temperature springs (US and DT) are relatively similar, except for the concentrations of fluoride, CaCO_3_, magnesium, and TN. On the other hand, the water of two low-temperature hot springs (SE and SS) differs in the concentrations of iron, phosphate, TN, and acidity. Additionally, the sulfur content in AH, US, and SK is relatively high, probably associated with their location in the northern part of Peninsular Malaysia. The TN content in SK and SE is quite high, with 5.6 mg L^−1^ and 6.1 mg L^−1^, respectively, while it is <0.2 mg L^−1^ for US and AH. Water in DT, SS, and SE has a carbon-to-nitrogen ratio (C:N) of 0.1–0.3, while the C:N for US and SK is quite similar (1.5–1.6). Besides its high COD and BOD levels, AH water has a high C:N ratio of 5.

**Figure 2:**
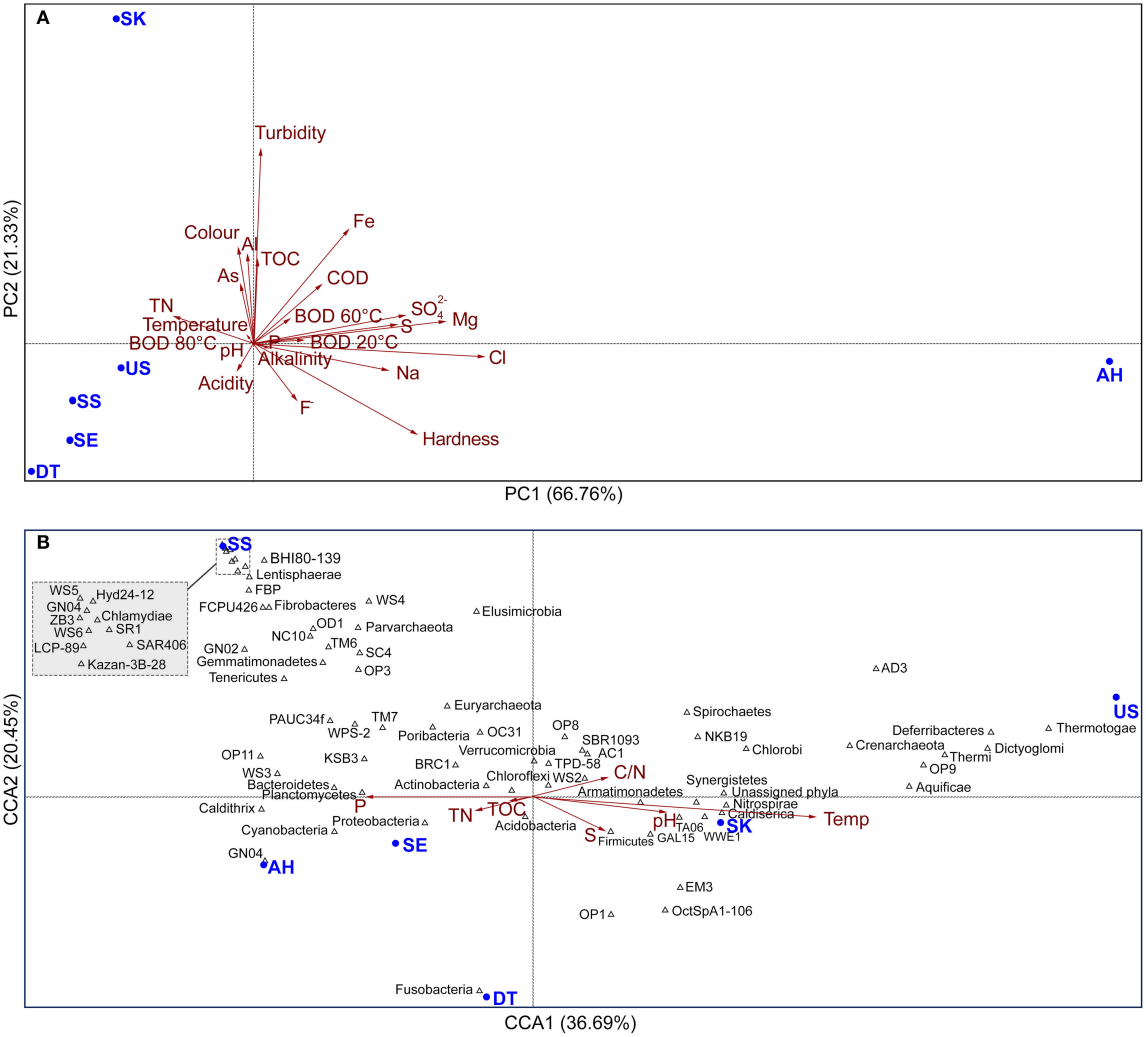
**(A)** PCA of physicochemical variables and **(B)** CCA of microbial phyla (>0.001% of total phyla sequences) in relation to physicochemical variables. The percent variability explained by each principal component is shown in parentheses in the axis labels. Each hot spring sample is represented by 1 filled circle. Red arrows indicate the direction and magnitude of physicochemical variables associated with microbial community structures. C:N, C:N ratio; temp, sampling-site water temperature; S, sulfur content; TN, total nitrogen; TOC, total organic carbon; and p, phosphate content. Different phyla are represented by phyla names and triangles.

PCA of the physicochemical variables separated the hot springs into three physicochemically distinct habitats (Group-1: AH; Group-2: SK, and Group-3: US, DT, SS, and SE). Figure 2A shows the distribution of the physicochemical variables formed by the first two components of the analysis, which explained 88.09% of the total variance. PC1 explained 66.76% of the observed variation, and clearly separated the saline hot spring AH from the non-saline hot springs (US, SK, DT, SS, and SE). PC1 was correlated with chloride, magnesium, sodium, sulfate ions, hardness, and sulfur, and high values for these variables might be related to the presence of seawater, as the AH hot spring is located on an island (Figure 1A). PC2, accounting for 21.33% of the total variation, was related primarily to pH, color, turbidity, aluminum, iron, and TOC. The relatively higher values of these variables in SK separated this hot spring from the other five springs; the presence of plant litter in SK increases the TOC (Chan et al., 2015). Group-3, a separate cluster of US, DT, SS, and SE, showed inverse correlations with most of the physicochemical variables, except temperature, TN, and acidity (Figure 2A).

### 16S rRNA gene microbial profiles

As summarized in Table 3, the sizes of the six 16S rRNA gene sequence datasets ranged from 0.60 to 2.32 Gb, with between 480,983 and 1,761,134 paired-end reads. After quality filtering, sequence merging, and chimera removal, more than 98.8% of the cleaned sequences were aligned against Greengenes database using the PyNAST (Caporaso et al., 2010a) alignment algorithm. After removal of the singletons (sequences that are present exactly once in a sample), 80.6–95.9% of the aligned sequences were taxonomically classified. About 4.1–19.4% of these sequences could not be assigned to known taxa, perhaps due to a lack of suitable reference sequences in the database. The majority of the assigned OTUs were classified as bacteria, i.e., 97.99% for US, 99.25% for SK, 99.48% for DT, 98.39% for SS, 99.51% for SE, and 99.99% for AH. Archaeal OTUs comprised relatively small percentages: 2.01% for US and 1.61% for SS, and <1% of the total populations for other sites (SK: 0.75%, DT: 0.52%, SE: 0.49%, and AH: 0.01%).

**Table 3:** Sequencing data profiles and alpha diversity indexes.

	**US**	**SK^a^**	**DT**	**SS**	**SE**	**AH**
Number of raw sequences	1,053,625	480,983	1,028,376	1,761,134	1,167,238	1,100,225
Average size read (bp)	35–301	35–301	35–301	45–301	35–301	35–301
Mean GC content (%)	58	56	54	53	54	55
High quality sequences^b^	1,013,171	429,677	952,470	1,650,101	1,084,632	1,057,048
Cleaned sequences^c^	990,519	424,188	914,072	1,627,045	987,849	984,566
PyNAST aligned sequences	987,974	421,383	912,827	1,608,109	985,204	982,466
Sequences without singletons	974,062	417,783	898,358	1,569,331	970,574	968,368
Total OTUs^d^	7,326	6,334	9,083	26,244	11,504	6,430
Taxonomy assigned sequences	801,105	376,334	861,537	1,264,280	912,975	909,979
Bacteria	785,038	373,507	857,088	1,243,916	908,541	909,890
Archaea	16,067	2,827	4,449	20,364	4,434	89
Unassigned sequences	172,957	41,449	36,821	305,051	57,599	58,389
**ALPHA DIVERSITY ANALYSIS**						
Good's coverage	0.985	0.987	0.983	0.974	0.982	0.985
Shannon-Wiener	6.505	9.020	6.123	9.929	7.128	7.513
Simpson	0.962	0.992	0.933	0.984	0.955	0.977

a *Data collected from Bioproject accession number PRJEB7059*.

b *Sequences which passed the quality filtration and sequence read merging process*.

c *Sequences obtained after chimera removal*.

d *Amount of observed OTUs after singletons removal*.

### Taxonomic composition of the prokaryotic communities

#### Bacterial diversity

**Figure 3:**
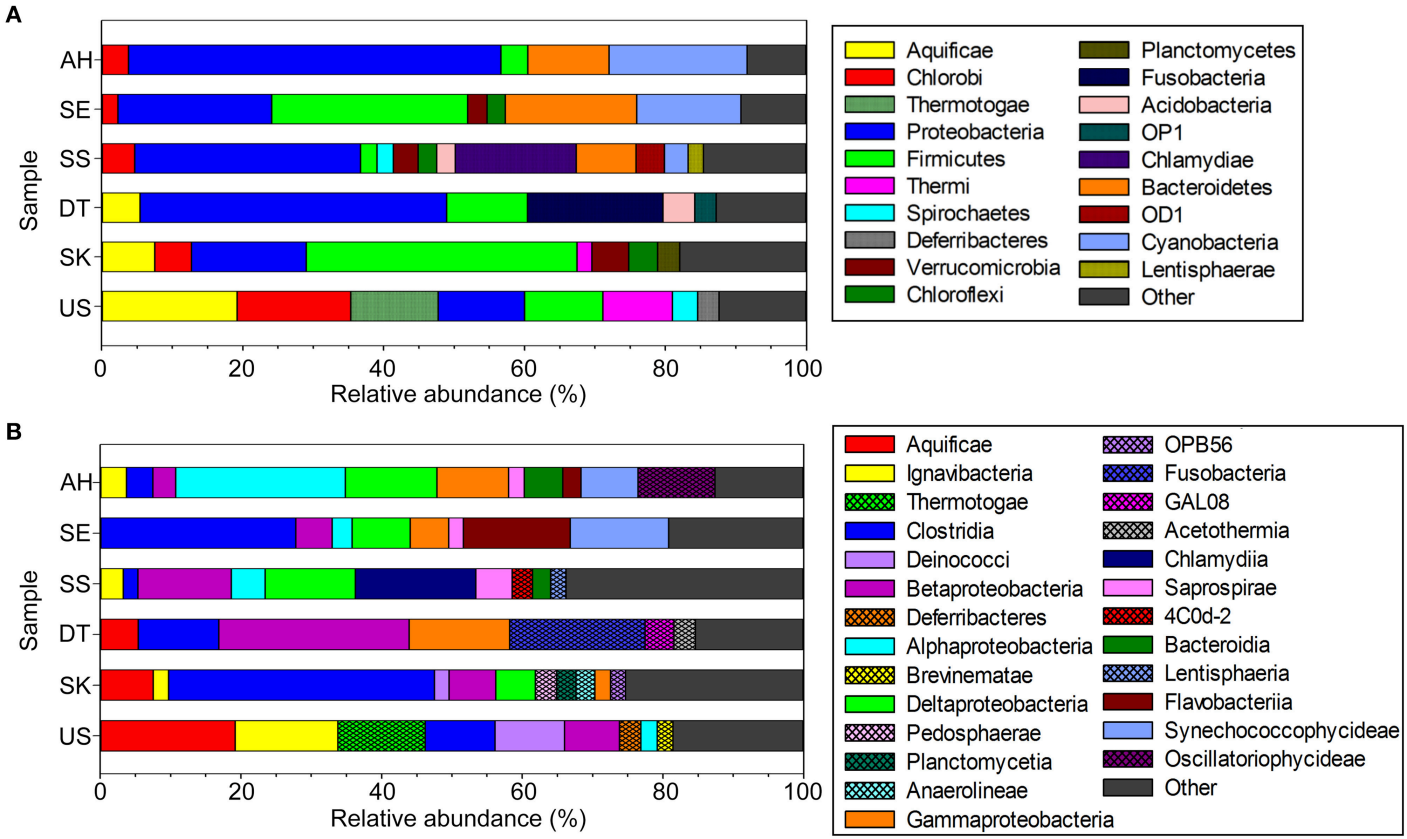
Relative abundance of bacterial 16S rRNA gene sequences. **(A)** Phyla and **(B)** class taxonomic levels of bacterial community structures with relative abundances of <2% were grouped as “Other.”

Within the domain Bacteria, more than 99.4% of the assigned sequences were classified at the phylum level. In total, 59, 61, 72, 73, 65, and 52 bacterial phyla were detected in US, SK, DT, SS, SE, and AH, respectively, yet less than six phyla are dominant in each hot spring with >10% relative abundance of total sequences at the phylum level. The bacterial communities within each site were unique. In general, the phyla Aquificae (relative abundance of 19.2%), Chlorobi (16.1%), Thermotogae (12.4%), Proteobacteria (12.2%), and Firmicutes (11.1%) dominated US. Phyla Firmicutes (38.5%) and Proteobacteria (16.3%) dominated SK, while Proteobacteria (43.5%), Fusobacteria (19.3%), and Firmicutes (11.5%) dominated DT. The SS hot spring was dominated by Proteobacteria (32.1%) and Chlamydiae (17.2%). SE water samples had great proportions of Firmicutes (27.8%), Proteobacteria (21.8%), Bacteroidetes (18.7%), and Cyanobacteria (14.8%). AH was dominated with Proteobacteria (52.8%), Cyanobacteria (19.6%), and Bacteroidetes (11.5%) (Figure 3A).

Firmicutes can be regarded as a signature phylum for circumneutral hot springs based on their abundance. Among the studied sites, SK and SE have the greatest dominance of Firmicutes (38.5 and 27.8% of total phyla, respectively), followed by US and DT (~11%), while this phylum is relatively less abundant in SS and AH hot springs (<4%). In general, at least 89.6% of total Firmicutes OTUs were assigned to the class Clostridia (mainly in the order Clostridiales, comprising obligate anaerobe and endospore-forming bacteria) (Figure 3B), while the remaining classes were Bacilli and Erysipelotrichi. In SK and SE, Firmicutes were mainly represented by the genera *Pelosinus* (15.4% of total Firmicutes) and *Acidaminobacter* (74.7%). Furthermore, Firmicutes in SE were dominated by the genus *Acidaminobacter*, with 37.8% of the total genera.

Proteobacteria form another signature phylum as the phylum was dominant in DT, SS, and AH (43.5, 32.1, and 52.8% of total phyla, respectively). High abundance of Proteobacteria was also detected in the US (12.2%), SK (16.3%), and SE (27.8%) hot springs. Betaproteobacteria form the most abundant Proteobacteria class in US, SK, DT, and SS, while AH was dominated by Alphaproteobacteria (Figure 3B). Moreover, Alpha-, Beta-, Gamma-, and Deltaproteobacteria were detected in all six Malaysian hot springs, but at different percentages. DT was dominated by *Vogesella* (43.3% of total genera), a genus within Betaproteobacteria. *Vogesella* is frequently found in freshwater bodies and currently, seven species have been described in this genus. *Vogesella lacus* (Chou et al., 2009) and *Vogesella perlucida* (Chou et al., 2008) are the only species that can grow at temperatures <40°C. The genus *Hahella* represented the main Gammaproteobacteria in AH (13.9%). *Hahella* are marine bacteria that require NaCl to grow. To date, only three species within the genus *Hahella* have been described, i.e., *H. chejuensis* (Lee et al., 2001), *H. ganghwensis* (Baik et al., 2005), and *H. antarctica* (Lee et al., 2008). *H. chejuensis* is able to grow at temperatures of up to 45°C (Lee et al., 2001).

Owing to its high water temperature (80–110°C), hyperthermophilic phyla including Aquificae and Thermotogae prevailed in US. Both phyla were also detected in SK (7.5% Aquificae and 1.7% Thermotogae) which has a considerable high temperature, but were insignificant in SS, SE, and AH (<0.1% of total phyla). Aquificae in US and SK were mainly comprised of the genus *Hydrogenobacter*, with 98.9% in US and 88.6% in SK of total Aquificae, equivalent to 31.6 and 13.3% of total OTUs at the genus level, respectively. With an average temperature of 75°C, in DT, Aquificae (5.4% of total phyla) were more abundant than Thermotogae (0.2%).

#### Archaeal diversity

**Figure 4:**
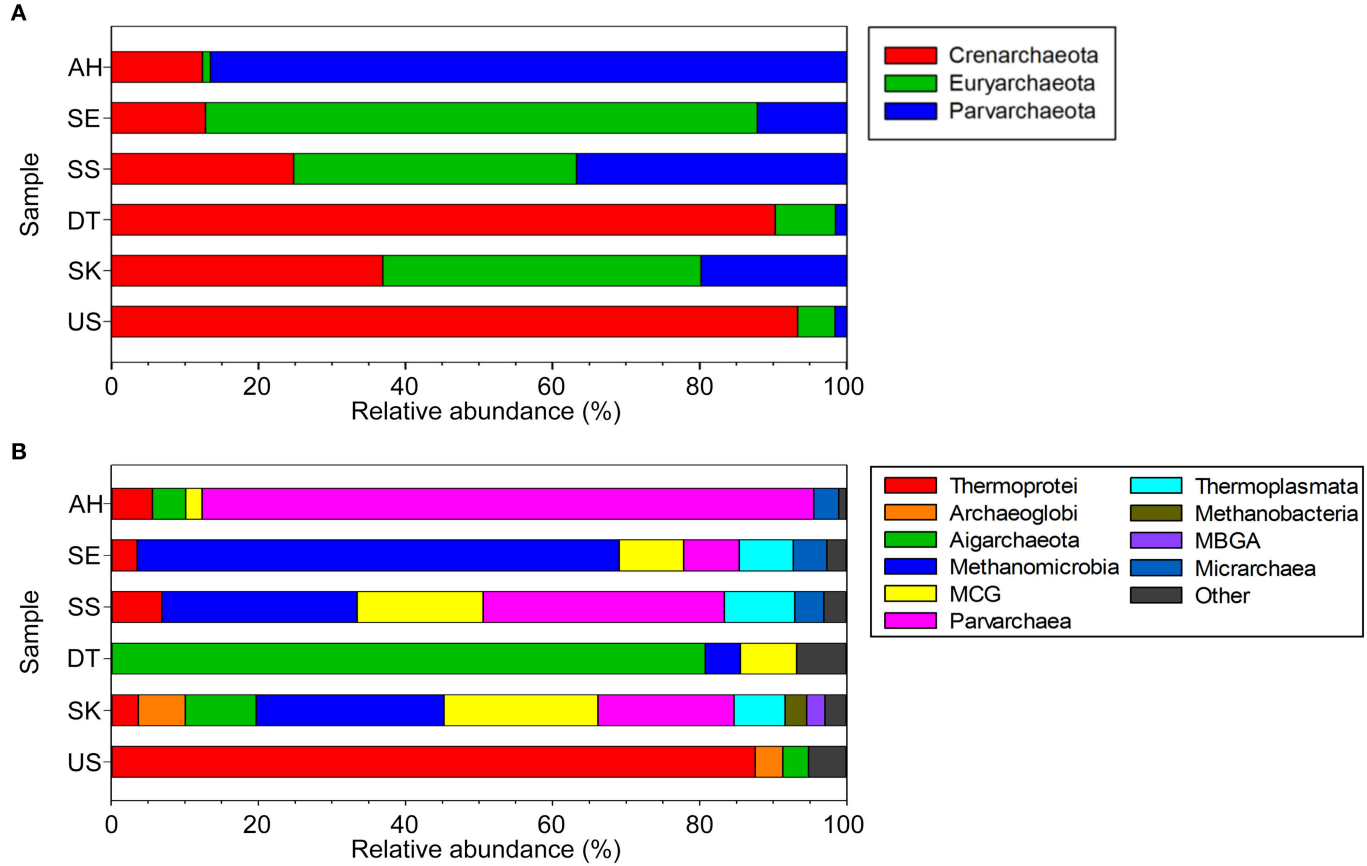
Relative abundance of archaeal 16S rRNA gene sequences. **(A)** Phyla and **(B)** class taxonomic levels of archaeal community structures with relative abundances of <2% were grouped as “Other.” Relative abundance is defined as the percentage of sequences in total successfully assignable sequences in the samples using Greengenes database.

Only 3 archaeal phyla, Euryarchaeota, Parvarchaeota, and Crenarchaeota, were detected in the studied sites (Figure 4A). Euryarchaeota were found to be the main archaea in SK (43.3% total archaea phylum), SS (38.5%), and SE (75.1%). Parvarchaeota prevailed in AH, with 86.5% of total archaeal phyla. An earlier metagenomics study elucidated that members of Parvarchaeota are very small in size (cells are <500 nm in diameter) and are from lineages without cultivated representatives that branch near the crenarchaeal/euryarchaeal divide (Baker et al., 2010). In SK, SS, and SE hot springs, the majority of the Euryarchaeota were methanogenic archaea of the class Methanomicrobia, genus, *Methanosaeta*. At present, *Methanosaeta* is the only genus affiliated to the Methanosaetaceae family. *Methanosaeta* spp. utilize acetate as a sole energy source to produce methane and carbon dioxide (Welte and Deppenmeier, 2011).

The phylum Crenarchaeota constituted the major archaeal member in the US (93.3%) and DT (90.3%) hot springs. In US, 93.7% of total Crenarchaeota were related to the class Thermoprotei. The hyperthermophilic genus *Aeropyrum* (90.4% of total genera) was identified in US; strains of this genus grow optimally at 85–95°C (Sako et al., 1996; Nakagawa et al., 2004). In DT (Figure 4B), 55% of the detected Aigarchaeota showed the closest similarity to the uncultivated archaeon “*Candidatus* Caldiarchaeum” (Nunoura et al., 2011). *Ca*. C. subterraneum was first discovered in a non-cultured metagenomic library from a microbial mat at a geothermal water stream of a sub-surface gold mine with a temperature of 70°C (Hirayama et al., 2005). It was proposed that *Ca*. C. subterraneum lives symbiotically with acetogenic “*Candidatus* Acetothermus autotrophicum” for organic carbon supply, since *Ca*. C. subterraneum has an extremely poor carbon fixation potential (Takami et al., 2015).

### Alpha diversity analysis

Since rarefaction curves did not reach a plateau (Supplementary Figure 1), total species richness was not estimated. Therefore, Good's coverage estimator was used; all six hot spring samples had an estimated coverage of at least 97% of the entire sampled population (Table 3). Additionally, non-phylogeny-based metrics including Shannon–Wiener's and Simpson's diversity indexes were computed (Table 3). A greater (Shannon–Wiener) diversity of OTUs was found in SS (9.9) and SK (9.0), followed by AH (7.5) and SE (7.1). Both US (6.5) and DT (6.1) samples have relatively lower diversity, based on both the Shannon–Wiener and Simpson indexes. It has been reported that high temperature has a negative effect on diversity (Sharp et al., 2014; Li et al., 2015). The microbial communities in high-temperature hot springs (US and DT, >70°C) were dominated by fewer genera than those in low-temperature sites (SS, AH, and SE, <50°C). Nevertheless, although the SK hot spring has a high water temperature of 50–110°C, the diversity index for SK is high.

### Beta diversity analysis

**Figure 5:**
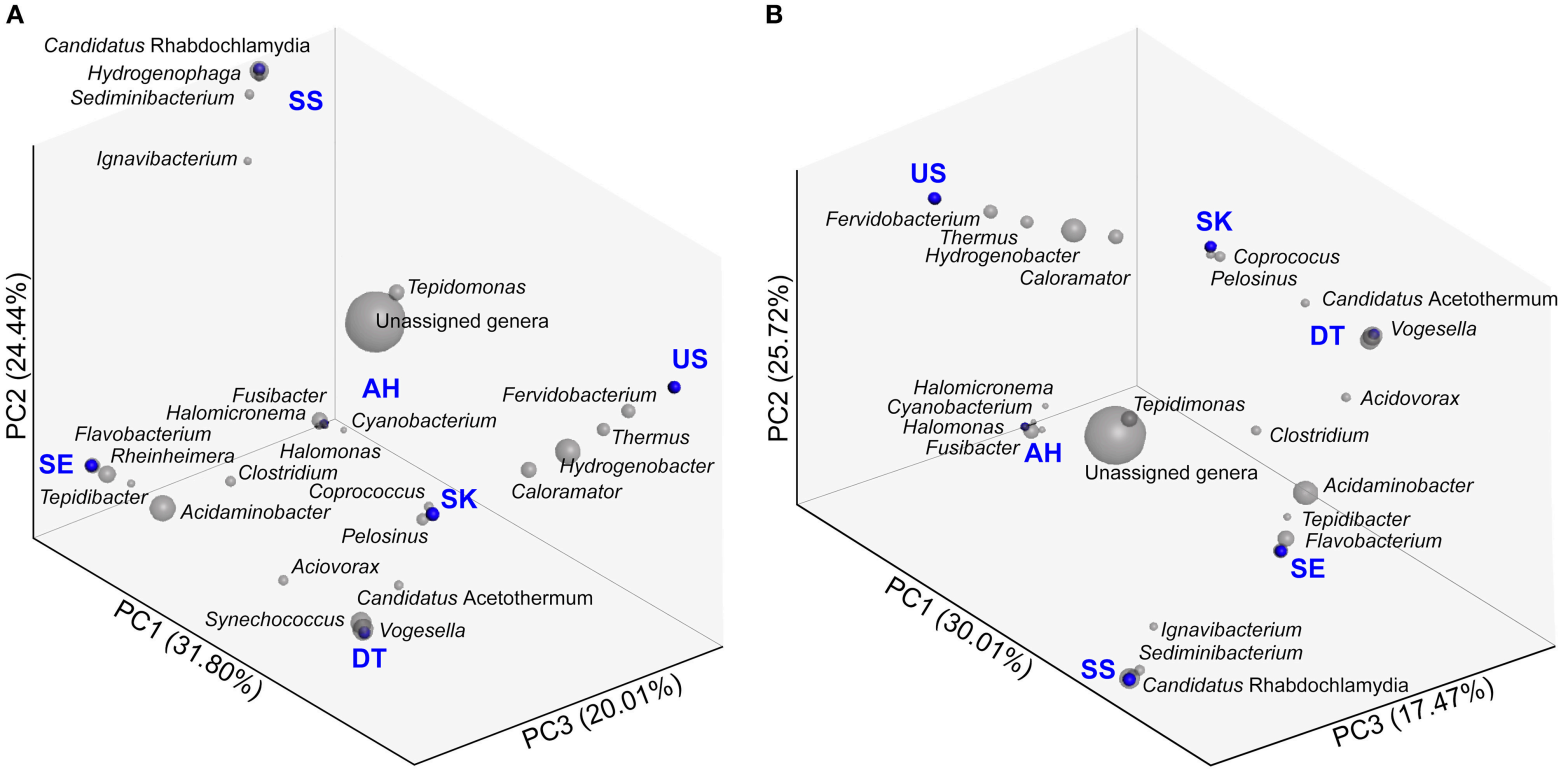
Communities clustered using PCoA of the **(A)** weighted and **(B)** unweighted UniFrac distance matrixes. PC1, PC2, and PC3 are plotted on x-, y-, and z-axes with the percentage of variation explained by each axis noted in parentheses. Each point corresponds to a hot spring community.

The above alpha diversity metrics provided an overview of the microbial diversity of each sampling site in this study. On the other hand, beta diversity is useful for documenting the structure of communities that may occur between samples categories or across environmental gradients (Lozupone and Knight, 2005). Here, we compared the six Malaysian hot spring samples using jackknifed UPGMA to cluster the community samples with weighted and unweighted UniFrac phylogenetic distances (Lozupone and Knight, 2005; Lozupone et al., 2007). Using PCoA with weighted UniFrac (Figure 5A), PC1 explains 32% of the variation, while PC2 and PC3 explain 24 and 20%, respectively. The AH hot spring presented as an outlier in PCoA owing to its high salinity. Weighted UniFrac PCoA and the corresponding dendrogram (Supplementary Figure 2A) were unable to show the effect of temperature on the microbial communities in the six hot springs. In comparison, PCoA with unweighted UniFrac (73% of total variation explained by PC1, PC2, and PC3) (Figure 5B) and the corresponding dendrogram (Supplementary Figure 2B) yielded a clearer clustering that sorted the hot springs into three groups in accordance to variation in temperature and salinity. The three groups are: Group-1: high salinity with moderate temperature (AH); Group-2: low salinity with moderate temperature (SE and SS); and Group-3: low salinity with high temperature (US, SK, and DT). A similar grouping pattern was identified based on Bray–Curtis dissimilarity using the jackknifed UPGMA dendrogram (Supplementary Figure 2C).

### Relationships between microbial community and physicochemical variables

Multivariate analyses are widely used in ecology studies to elucidate the relationships between abundances of certain organisms and environmental parameters. In this study, the CCA approach (ter Braak and Verdonschot, 1995) was used to identify possible relationships between microbial communities in hot springs and local physicochemical variables. Only physicochemical variables with normally distributed values, including temperature, pH, C:N, TOC, TN, sulfur, and phosphate, were used in this study (Figure 2B). The placement of hot spring samples in the plot is influenced by their environmental characteristics. The eigenvalues for each axis generated by CCA indicate how much of the variation seen in the genera data can be explained by the canonical axis. In this analysis, 61% of the correlation between OTUs, hot spring samples, and physicochemical variables were explained by two axes.

Most variables, including temperature, pH, C:N, and sulfur content showed a positive correlation (Figure 2B), while inverse correlations were observed for TOC, TN, and phosphate content. Among the tested variables, temperature was the most influential variable affecting the distribution of OTUs. In Figure 2B, plots for US and SK are situated in a right side, as these sites are characterized by high temperatures, while SS, AH, and SE hot springs fall in the opposite side of the plot. The right side was occupied by thermophilic phyla, including Thermotogae, Dictyoglomi, Thermi, Aquificae, Caldiserica, Crenarchaeota, Deferribacteres, candidate divisions OP9 (“Atribacter”) (Nobu et al., 2016), OP1, EM3, and OctSpA1-106 (Youssef et al., 2015), with a strong, positive correlation with temperature. Additionally, candidate divisions EM3, OP1, and OctSpA1-106 are also positively correlated with sulfur content, and these OTUs were frequently found in US, SK, and DT, which are rich in sulfur (Table 2). Furthermore, phosphate is positively correlated with Caldithrix, Bacteroidetes, Cyanobacteria, Proteobacteria, WS3, and OP11, which were found in the AH and SE hot springs with slightly higher phosphate contents. Proteobacteria, Cyanobacteria, and Bacteroidetes were also positively correlated with TOC and TN, while Chlorobi, candidate division NKB19, and Spirochaetes were positively correlated with C:N. The microbial community from SS was negatively correlated with most of the measured physicochemical variables.

## Discussion

### Microbial community composition and diversity

All reported Malaysian hot springs have circumneutral pH. In the present study, the distributions of microbiota in six hot springs with different physical and physicochemical characteristics were studied. The five non-saline hot springs can be differentiated based on their water temperature, i.e., moderate-temperature (SS and SE) and high-temperature (US, SK, and DT) thermal springs. The moderate-temperature hot spring AH is especially interesting, as it is located 2 km away from an open sea, on an island. The concentrations of sodium, magnesium, chloride, fluoride, and sulfate ions in the AH hot spring were nevertheless lower than those in a seawater sample collected from Malaysia (data not shown). Therefore, it is possible that the water in the AH pond is a mixture of sea- and groundwater. The compounds in seawater (including chloride, magnesium, sodium, and sulfate ions) clearly influenced the microbial community that distinguishes AH from the non-saline US, SK, DT, SS, and SE hot springs. The microbial community structures in the US and SK hot springs are quite similar; both sites cluster together in the biplots in Figures 5A,B and share similar OTUs such as *Thermus, Hydrogenobacter*, and *Caloramator*.

Generally, the dominant phyla in the studied hot springs are similar, but the sites differ with respect to the overall composition (Figures 3A, 4A). Firmicutes and Proteobacteria are the phyla consistently present in circumneutral hot springs. Site-specific taxa assigned at the genus level included *Fervidobacterium* in US; *Coprococcus* and *Pelosinus* in SK; *Vogesella* in DT; *Candidatus* Rhabdochlamydia, *Sediminibacterium*, and *Hydrogenophaga* in SS; *Rheinheimera* and *Flavobacterium* in SE; and *Halomicronema, Halomonas, Fusibacter*, and *Cyanobacterium* in AH (Figure 5A). Interestingly, *Candidatus* Rhabdochlamydia (Kostanjšek et al., 2004), an intracellular bacterium that was first found in the terrestrial isopod *Porcellio scaber* appeared to be one of the dominant genera in SE. This finding suggests that endosymbiosis of thermophilic microbiota can occur in the hot springs and is not restricted to SE, but also possibly occurs in DT, where we discovered tiny reddish crustaceans (order, Isopoda; data not shown).

In the current study, archaea appeared to be a minority in the prokaryotic community. This result is consistent with our previous shotgun metagenome analyses for SK (Chan et al., 2015) and US (data not shown). High-temperature environments were previously generally believed to be the realm of archaea (Urbieta et al., 2014; Li et al., 2015). However, recent studies applying molecular methods have revealed that bacteria rather are the predominant prokaryotic communities in such environments (Badhai et al., 2015; López-López et al., 2015). The factors that allow bacteria to dominate in high-temperature habitats are not well understood. Our findings revealed that archaea are not dominant in circumneutral hot springs, which in agreement with several recent reports with similar pH ranges (Wang et al., 2013; Merkel et al., 2017). Though insightful, the above findings are preliminary, as they are based on molecular methods, which inherently assume that both bacteria and archaea are detected at the same level. Moreover, other technical factors including DNA extraction method (Zielińska et al., 2017), primer selection (Cai et al., 2013), primer combinations, library preparation protocols, and sequencing platforms should be considered.

Higher Shannon–Wiener or Simpson diversity index values indicate greater species richness. The moderate-temperature springs SS, SE, and AH were more diverse than the higher-temperature springs at US and DT (Table 3). This shows that increments in habitat temperature result in decreased taxonomic richness and diversity, which is in agreement with earlier findings (Miller et al., 2009; Tobler and Benning, 2011; Inskeep et al., 2013; Sharp et al., 2014). Yet, markedly high microbial richness and diversity exist in SK, which may be owing to the stream-like physical appearance of this hot spring. Along the heated stream, the pH gradient varies by 1.5 unit, and the temperature fluctuates (60–110°C) because of the presence of multiple spring heads. Moreover, the stream is shallow and the water flows rapidly, thus generating sufficient aeration for aerophiles. Finally, the presence of plant litter in SK is associated with high TOC (additional carbon source) (Hou et al., 2013), which favors the growth of microorganisms. Thus, fluctuations in physicochemical features in a microenvironment likely enable a wider range of microbial species to survive.

### Physicochemical factors regulating microbial community structure

Quantitative weighted UniFrac analysis (Figure 5A) of our samples suggested that the five non-saline hot springs had similar microbial communities, while AH represented an outlier in the dendrogram (Supplementary Figure 2A). The high salinity of AH should be the main factor responsible for the unique microbial community based on the relative abundances of OTUs. Compared to quantitative weighted UniFrac, unweighted UniFrac is a qualitative distance metric that only considers the presence or absence of OTUs. Unweighted UniFrac further confirmed that microbial membership in the saline spring (AH) largely differs from that in the non-saline hot springs. Besides salinity, unweighted UniFrac showed clustering of the microbial community structures by temperature for the non-saline hot springs (Figures 5B and Supplementary Figure 2B). This explained that temperature is a crucial factor in identifying the changes in community membership rather than community composition in this study. Taken together, our data indicate that salinity and temperature are the main factors in shaping the microbial community structures in these six Malaysian hot springs.

Besides temperature and salinity as the most influential factors, we were interested in identifying other factors potentially affecting the microbiota in circumneutral hot springs. Based on the CCA plot, it is possible that TOC and TN, or the C:N ratio affect growth efficiencies, and thus shape the microbial communities (Michaud et al., 2014; Wan et al., 2015). AH has the highest C:N among the six Malaysian hot springs, partly because seawater has a high C:N (Meyers, 1994). DT, SS, and SE hot springs are more species-rich, and these sites have low C:N (<0.5) (Touratier et al., 1999). Even though DT, US, and SK are high-temperature springs, the dominant genera in DT are different from those in the other two springs, probably owing to low C:N ratio. As SK is located in a forest, it has a relatively high TOC, possibly related to the presence of plant litter along the stream. The decomposition of plant litter is one of the processes involved in nutrient and carbon cycling in ecosystems, and results in the release of dissolved organic matter back to earth (Kindler et al., 2011). The high quantity of organic matter in SK might explain the high microbial diversity and richness of this hot spring in comparison to other springs.

CCA showed a positive correlation between phosphate content and phosphate-solubilizing bacteria from phyla Proteobacteria, Actinobacteria, and Bacteroidetes (Sharma et al., 2013). This suggests that Cyanobacteria, Planctomycetes, Caldithrix, candidate divisions OP11, and WS3 are likely involved in the process of releasing phosphorus from insoluble compounds to the environment. Additionally, the negative correlation between Cyanobacteria and water temperature was shown for high-temperature springs (US, SK, and DT hot springs). Such observation is explained by the restriction of photosynthesis when the temperature is higher than 75°C (Ferris and Ward, 1997). In another aspect, the positive correlation between sulfur content and candidate divisions OP1, EM3, and OctSpA1-106 suggests that sulfur may affect the growth of these uncultured prokaryotes. These candidate divisions may be important for sulfur cycling in hot springs. In comparison to aforementioned variables, other abiotic factors, including chloride, fluoride, sodium, sulfate, iron, magnesium, arsenic, and aluminum play less of a role in determining the predominant microbial members in the Malaysian hot springs.

## Conclusion

The six Malaysian hot springs in this study have different physical and physicochemical characteristics. Investigating hot spring microbiomes with simple microbial composition is important for understanding microbe-mediated biogeochemical cycles and ecosystem functioning. This is the first study to identify the physicochemical factors that drive variations in microbial community structures in Malaysian hot springs. Firmicutes and Proteobacteria were the signature phyla in all six hot springs that along with the presence of site-specific taxa contributed to the uniqueness of each hot spring. Temperature was found to be the most influential factor shaping the microbiome of Malaysian hot springs, as was anticipated. Generally, overall microbial diversity and richness were negatively affected by temperature. As an exception, SK hosted high microbial richness and diversity despite its high temperature, probably due to its physical characters that enable a wider range of microbial species to survive. Variables such as salinity, C:N ratio, phosphate, and sulfur content are probably secondary factors that affect circumneutral hot spring microbial communities. Nevertheless, other variables should not be ignored in microbial ecology studies, as all abiotic factors collectively contribute to the dynamics of microbial populations. Understanding microbial community dynamics and genomic variability of community members in hot springs with different ecologies is important to elucidate community functions and their importance for the maintenance of hot spring ecosystems.

## Author contributions

CC, KC, and KG contributed to the conception and design of the study. CC, RE, and KH produced data. CC, KH, MS, and KG conducted the bioinformatics and statistical analyses. CC, KC, and KG wrote and reviewed the manuscript. MU, ED, and MS helped in interpretation of data and contributed to the discussion of the results followed by reviewing the manuscript. All authors read and approved the final manuscript, and are agreement to be accountable for all aspects of the work in ensuring that questions related to the accuracy or integrity of any part of the work are appropriately investigated and resolved.

### Conflict of interest statement

The authors declare that the research was conducted in the absence of any commercial or financial relationships that could be construed as a potential conflict of interest.

## Footnotes

^1^http://www.1000springs.org.nz/

^2^http://hannonlab.cshl.edu/fastx_toolkit

## Abbreviations

AH: Ayer Hangat
BOD: biochemical oxygen demand
CCA: canonical correspondence analysis
C:N: carbon-to-nitrogen ratio
COD: chemical oxygen demand
DT: Dusun Tua
OTU: operational taxonomic unit
PCA: principal component analysis
PCoA: principal coordinate analysis
QIIME: Quantitative Insights Into Microbial Ecology
SE: Semenyih
SK: Sungai Klah
SS: Sungai Serai
TN: total nitrogen
TOC: total organic carbon
UPGMA: unweighted pair group method with arithmetic mean
US: Ulu Slim.
