# Supplementary material for: Effects of Physiochemical Factors on Prokaryotic Biodiversity in Malaysian Circumneutral Hot Springs
Source: Front Microbiol. 2017 Jul 6;8:1252. doi: 10.3389/fmicb.2017.01252 (PMC5498555; doi:10.3389/fmicb.2017.01252)
Supplement: Supplementary file 1 [file Image1.PDF]

frontiers

Supplementary Material

Effects of Physiochemical Factors on Prokaryotic Biodiversity in Malaysian Circumneutral Hot Springs

Chia Sing Chan, Kok-Gan Chan, Robson Ee, Kar-Wai Hong, María Sofía Urbieta, Edgardo Rubén Donati, Mohd Shahir Shamsir, Kian Mau Goh*

*** Correspondence:** Kian Mau Goh: gohkianmau@utm.my

Supplementary Figures

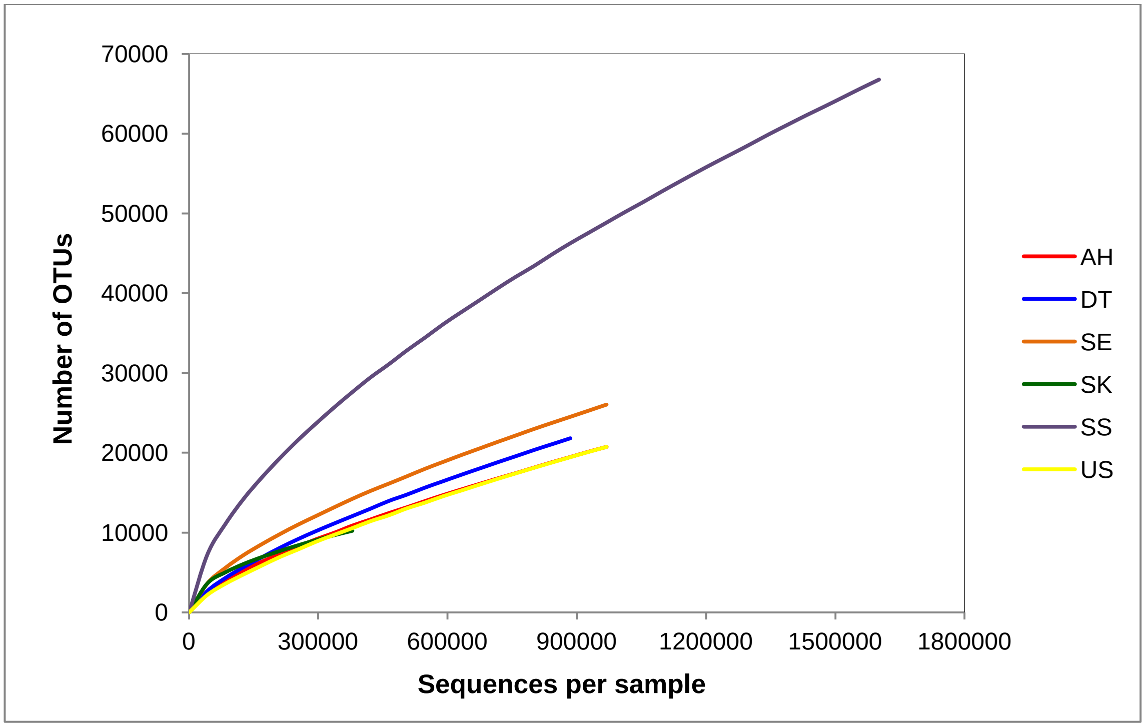

Supplementary Figure 1. Rarefaction analysis of observed OTUs among the six Malaysian hot springs.

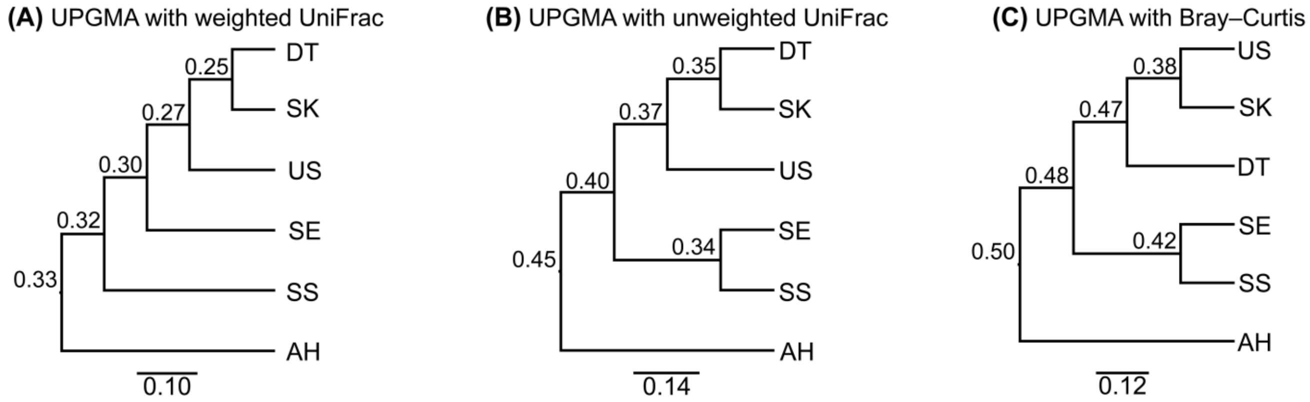

Supplementary Figure 2. UPGMA clustering of the six hot spring samples based on **(A)** weighted and **(B)** unweighted UniFrac, and **(c)** Bray–Curtis dissimilarity. The numbers associated with the branches refer to confidence coefficient where 1 represents maximal support, while the scale bars refer to amount of changes.
